# Regulation of Electromagnetic Perceptive Gene Using Ferromagnetic Particles for the External Control of Calcium Ion Transport

**DOI:** 10.3390/biom10020308

**Published:** 2020-02-15

**Authors:** Jangsun Hwang, Yonghyun Choi, Kyungwoo Lee, Vijai Krishnan, Galit Pelled, Assaf A. Gilad, Jonghoon Choi

**Affiliations:** 1 School of Integrative Engineering, Chung-Ang University, Seoul 06974, Korea; isnickawesome@gmail.com (J.H.); dydgus5057@gmail.com (Y.C.); orztapa@cau.ac.kr (K.L.); 2 Department of Biomedical Engineering, Michigan State University, East Lansing, MI 48823, USA; krish102@msu.edu (V.K.); pelledga@msu.edu (G.P.); 3 Department of Radiology, Michigan State University, East Lansing, MI 48823, USA

**Keywords:** synthetic biological device, electromagnetic perceptive gene, *Kryptopterus bicirrhis*, calcium ion, magnetic particles

## Abstract

Developing synthetic biological devices to allow the noninvasive control of cell fate and function, in vivo can potentially revolutionize the field of regenerative medicine. To address this unmet need, we designed an artificial biological “switch” that consists of two parts: (1) the electromagnetic perceptive gene (EPG) and (2) magnetic particles. Our group has recently cloned the EPG from the *Kryptopterus bicirrhis* (glass catfish). The EPG gene encodes a putative membrane-associated protein that responds to electromagnetic fields (EMFs). This gene’s primary mechanism of action is to raise the intracellular calcium levels or change in flux through EMF stimulation. Here, we developed a system for the remote regulation of [Ca^2+^]_i_ (i.e., intracellular calcium ion concentration) using streptavidin-coated ferromagnetic particles (FMPs) under a magnetic field. The results demonstrated that the EPG-FMPs can be used as a molecular calcium switch to express target proteins. This technology has the potential for controlled gene expression, drug delivery, and drug developments.

## 1. Introduction

Various methods of expressing a target protein using external stimuli have been studied. It is possible to express the desired protein via plasmid transfection [1] or viral transduction [2] with a magnetic field [3,4] and other methods [5,6]. Target proteins have been quantitatively expressed by external stimuli such as the control of substrate (isopropyl β-d-1-thiogalactopyranoside; IPTG) [7], temperature [8], nutrients [9], oxygen [10], and growth factors [11]. However, these controls take advantage of allosteric regulators, temperature, and growth factors used for normal protein expression. These are not the methods of expressing target proteins by active control that can be turned on or off by an external magnetic field. Various methods have been developed to express proteins at a desired period of time, using external- stimulation techniques [6,12,13,14,15,16]. The photostimulation method focuses on neuronal differentiation and proliferation wherein using optimal conditions of flash optical stimulation lead to a significant increase in the number of cell nuclei and neurons differentiated on nanostructures [17]. It has been suggested that plasmonic excitation of gold nanoparticles can be used to stimulate and monitor localized Ca^2+^ ion signaling in neurons. Nanoparticle assisted localized optical stimulation (NALOS) serves to be a valuable addition to the existing repertoire of techniques that involve light dependent control of neuronal activity [18]. However, methods that involve optical stimulations have limitations in tissue penetration. Therefore an alternative method that involves using a biological on/off switch as stimulus is desirable [14,19]. 

Ca^2+^ ions are usually present at a higher concentration, 10,000-fold or more, on the outside of the cell than the inside and are introduced into the cell by various mechanisms [20,21,22,23]. The opening and closing of Ca^2+^ ion channels are primarily regulated by differences in membrane potential [24,25,26,27,28] and different types of Ca^2+^ ion channels (ligand gated, voltage gated) have been described in literature extensively [29,30,31]. Intracellular Ca^2+^ ions play a central role in the signal transduction of cells through conjugation with calcium-binding proteins. The functions of Ca^2+^ ions are varied in neurons. These include secretion and regulation of neurotransmitter and neurohormones, cell growth and differentiation, immune cell activity, increases in the heart rate of cardiac cells, and increases in blood pressure due to vasoconstriction [24,25,26,27,28]. Therefore, developing a novel technique (biological on/off switch) to control intracellular calcium ions, would enable us to use it in cell-based studies and therapeutic applications.

This study utilizes the electromagnetic perceptive gene (EPG) that was isolated from *Kryptopterus bicirrhis* (glass catfish) and was demonstrated to respond to electromagnetic fields (EMF) [32]. Previous results have shown that EPG gene activation by an electromagnetic stimulus is primarily associated with an increase in intracellular calcium, [Ca^2+^]_i_ Activation of EPG by electromagnetic stimulus has been successfully reported in in-vivo (rodent) and in-vitro models (HEK cells, primary cortical neurons). However, the structure and function of the EPG gene has not been completely resolved.

In order to engineer an artificial bio-circuit that serves to target protein expression from the exterior of the cell, an integrated circuit consisting of a switch, transistor and a reporter is necessary. We designed a biological circuit, capable of controlling calcium migration into the target cells expressing EPG. The magnetic field was locally applied to EPG-expressing cells using ferromagnetic particles (FMPs) that could be used as a biological on/off switch to induce EPG expression, thereby enabling the target protein expression to be turned on and off. Magnetic field activation of target cells using FMPs led to an increase in [Ca^2+^]_i,_ as observed in real time [Ca^2+^]_i_ imaging experiments. It is expected that this system can be applied to various studies that involve promoter amplification by Ca^2+^ ions, cytokine secretion of immune cells, control of cell differentiation and to studycorrelations between calcium ions and contraction/relaxation of smooth muscle cells.

## 2. Materials and Methods

### 2.1. Materials

Streptavidin-coated ferromagnetic particles (permanent magnetic particles, FMPs, 2–2.9 µm) and streptavidin-coated superparamagnetic particles (no magnetism, SMNPs, 0.1–0.39 µm) were purchased from Spherotech, Lase Forest, IL, USA. SMNPs have no net magnetism at room temperature due to the Néel relaxation time. When there is an absence of an external magnetic field, the net magnetization would be zero. This would be referred to as the “off” state. The sulfo-NHS-biotin (*N*-Hydroxysulfosuccinimidobiotin) labeling kit, Lipofectamine 2000, Opti-MEM, Fura-2-AM, Pluronic F-127, well plates, and glassware were purchased from Thermo Fisher Scientific (Waltham, MA, USA). All reagents were dissolved in a calcium imaging solution (CIS). The CIS was prepared by mixing 125 mM NaCl, 2 mM MgCl_2_, 4.5 mM KCl, 10 mM glucose, 20 mM HEPES, and 2 mM CaCl_2_ adjusted to pH 7.4. The CIS was used for all Ca^2+^ imaging experiments.

### 2.2. Cell Culture

Human embryonic kidney 293T cells (HEK293Ts) were used for imaging experiments. The cell culture media consisted of the Dulbecco’s Modified Eagle’s Medium (DMEM) supplemented with 1% penicillin/streptomycin, 10% fetal bovine serum, and 5% CO_2_ at 37 °C. Subsequently, 1 × 10^5^/mL cells were seeded onto a glass-bottom dish and incubated for 24 h. 

### 2.3. Characterization of Magnetic Particles

FMPs [33] and SMNPs [34] were characterized by both transmission electron microscopy (TEM) and high-resolution TEM (HRTEM) images were obtained using a JEOL JEM-2100F, Tokyo, Japan. Samples were prepared by casting a drop of particle dispersion onto a carbon-coated copper grid. The room-temperature magnetization curves were obtained by a temperature magnetization curves were obtained by a Quantum Design PPMS-9 magnetomete, San Diego, CA, USA.

### 2.4. EPG Transfection

HEK293T cells were transfected with pcDNA3.1-EPG+GFP and Lipofectamine 2000 in a glass-bottom dish according to standard protocols. Transfected cells were incubated for 48–60 h at 37 °C, and GFP-expressing cells were confirmed by fluorescence microscopy.

### 2.5. Biotin Conjugated HEK293T^EPG+GFP^ Cells

Briefly, 1 mL of 0.1 mM sulfo-NHS-biotin in PBS was applied to the HEK293T^EPG+GFP^ cells and maintained for 30 min at 25 °C. NHS-activated biotins react efficiently with primary amine groups (-NH_2_) available on the surface proteins of HEK293T cells, that leads to streptavidin-biotin conjugation chemistry. After the reaction, cells were carefully washed twice with the CIS and labelled with Fura-2-AM. Before calcium imaging, 10 µL streptavidin-coated FMPs (1% *w*/*v*, 1 × 10^9^/mL) and SMNPs (0.5% *w*/*v*, 1.5 × 10^13^/mL) were added to the HEK293T^EPG+GFP^ for 10 min by using a silicon tubing system. Later, unbound magnetic particles were removed by washing.

### 2.6. Calcium Imaging in HEK293T^EPG+GFP^ Cells

Calcium imaging was performed on the HEK293T cells transfected with pcDNA3.1-EPG+GFP using Lipofectamine 2000 Reagent (Life Technologies, Inc., Carlsbad, CA, USA). The cells were used for imaging at 48 h post-transfection, once the maximum transfection efficiency was observed at this time point. The cells were subsequently washed three times with the CIS. The cells were loaded with 1 μM Fura-2-AM for 45 min at 37 °C. Then, the transfected cells were washed three times with the CIS and Fura-2-AM in the cells was de-esterified for 30 min at 37 °C. Culture dishes were placed into customized imaging chambers. An inverted Olympus 1X71 (Olympus Corporation, Tokyo, Japan) microscope was used to measure the fluorescence intensity changes of Fura-2-AM. The signal intensity changes reflect changes in the intracellular calcium concentration in the cells due to magnetic stimulation. Subsequently, GFP-positive cells were randomly selected using Metafluor imaging software (Molecular Devices, San Jose, CA, USA) and changes in the [Ca^2+^]_i_ were recorded as ratiometric alterations in both 340 and 380 nm wavelengths. A magnetic stimulus was applied by using the FMPs or SMNPs that subsequently showed magnetic field strengths of ~200 and 0 µT at 100 s, respectively. The data were analyzed as changes in the ratiometric fluorescence intensity of Fura-2-AM over time, post magnetic stimulation.

### 2.7. Cell Identification

To identify responding cells (Responded cell; *R*), the fluorescence intensity of each individual cell was measured at 100 s prior to magnetic stimulation (*I_before_*). The maximum fluorescence intensity of an individually responding cell was also measured after magnet particle stimulation (IMafter). The mean value (100–103 s) of *I_before_* was the *I_mean_*.The mean value (200–223 s) of *IM_after_* is *IM_mean_* and the standard deviation before magnetic stimulation is abbreviated SD. The measured responded cells were described as a numeric value at a given time point. Responding cells were determined using the following equation (Equation (1)) [32]:

R = IM_mean_ > I_mean_ + (5 × SD). (1)

### 2.8. Cytotoxicity Tests

HEK293T cells were cultured for 24 h at 80% confluence in a 96-well plate (1 × 10^5^ cells/well). Each well was treated with different concentrations of FMPs (1% *w*/*v*, 1 × 10^9^/mL) or SMNPs (0.5% *w*/*v*, 1.5 × 10^13^/mL) for 24 h. Post treatment, the numbers of live and dead cells were counted via Trypan blue dye exclusion assay.

### 2.9. High-Resolution Fluorescent Microscopy Images

High-resolution fluorescent microscopy images of the cells were obtained using a Carl Zeiss LSM 710 laser-scanning microscope (Carl Zeiss, Oberkochen, Germany). HEK293T^EPG+GFP^ cells were fixed with 4% paraformaldehyde (PFA) for 30 min and permeabilized with 0.5% Triton X 100 for 60 min. Post fixation, cells were dried and washed twice with PBS, followed by blocking with a 2% bovine serum albumin solution for 2 h. HEK293T^EPG+GFP^ cells were stained with rabbit anti-EPG antibody (1:100 dilution) for 2 h followed by addition of donkey Alexa-595 conjugated anti-rabbit antibody (1:2000 dilution) with overnight incubation. Finally, a 1 mL (3nM) working solution of DAPI (4′,6-diamidino-2-phenylindole) was added to stain the nuclei of the fixed cells, followed by mounting and sealing.

### 2.10. Statistical Analysis

GraphPad Prism software was used for statistical analysis and graphical representations of data. Student’s *t* tests were performed on the data to evaluate significance. Non-significant values are shown as ns in the Results section, while *, **, and *** describe the *p* values of <0.05, <0.01, and <0.001, respectively.

## 3. Results and Discussion

**Figure 1:**
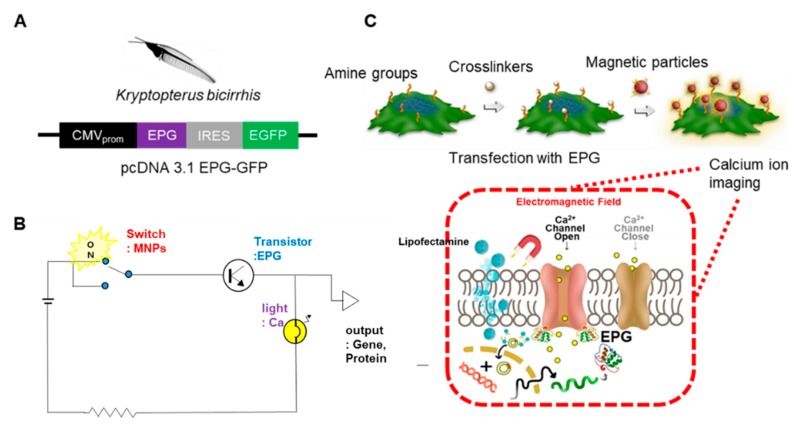
Illustration of the remote regulation of [Ca^2+^]_i_ in human embryonic kidney (HEK^EPG+GFP^) cells. (**A**) Plasmid encodes of both the electromagnetic perceptive gene (EPG) and the Green fluorescent protein (GFP) using Internal ribosome entry site (IRES) under the regulation of a general cytomegalovirus promotor (CMV promoter) were constructed. (**B**) Scheme of engineering biological circuit; switch = magnetic particles, transistor = EPG, light = Ca imaging, output = target gene/protein. (**C**) EPG+GFP was expressed in HEK293T. Sulfo-NHS-SS-Biotin was used to crosslink biotin with amine groups of cell-surface proteins. Streptavidin-coated Manetic particles (MPs) were added to the media and conjugated to biotin moieties on the cells (MPs are located outside of cells). [Ca^2+^]_i_ imaging was employed to monitor the cell activation profiles with magnetic stimulation. EPG is a transmembrane protein; the origin of the Ca ion before the magnetic stimulation of cells is unknown; Ca ion is mainly stored in Endoplasmic reticulum (ER) but the uptake of Ca ion should be considered.

The HEK293T cells were transfected with a pcDNA3.1 vector construct containing the EPG gene under the CMV promoter pcDNA3.1-EPG+GFP [32] (Figure 1A). Figure 1B shows the schematic drawing of the biological circuit. The switch is turned on (irreversible) by magnetic particles (FMPs) that generate a local magnetic field around the target cells. The switch is turned on (irreversible) by magnetic particles (FMPs) that generate a local magnetic field around the target cells. This results in the activation of EPG thereby providing signal amplification. Calcium imaging confirms the current flow represented as light in the circuit. The final result is the expression of the target genes/proteins in cells represented as output in the circuit. To evaluate this biological circuit platform with the EPG, we designed a remote regulation of [Ca^2+^]_i_ in the HEK293T^EPG+GFP^ cells. The amine functional groups of surface proteins on the HEK293T^EPG+GFP^ cells were individually conjugated with biotin molecules by using sulfo-NHS-SS-biotin crosslinkers. Subsequently, streptavidin-coated magnetic particles were added at fixed time points during Ca imaging with Fura-2-AM. This setup allowed the monitoring of [Ca^2+^]_i_ with the addition of FMPs or superparamagnetic nanoparticles (SMNPs) that serve as an on/off switch for the expression of the EPG and [Ca^2+^]_i_ (Figure 1C). The downstream pathways leading to subsequent increases in intracellular Ca is yet to be delineated. 

### 3.1. Characterization of Magnetic Particles

Transmission electron microscope (TEM) images confirmed the presence of FMPs and SMNPs (Supplementary Figure S1). The magnetic field strength of FMPs measured by the Gauss meter was 0.2 mT in 10 µL and was not detected in SMNPs (simply 10 µL of FMPs was dropped on the Gauss meter). This implies that the activation of the EPG was possible at as low as 0.2 mT by FMPs.

### 3.2. Expression of EPG

**Figure 2:**
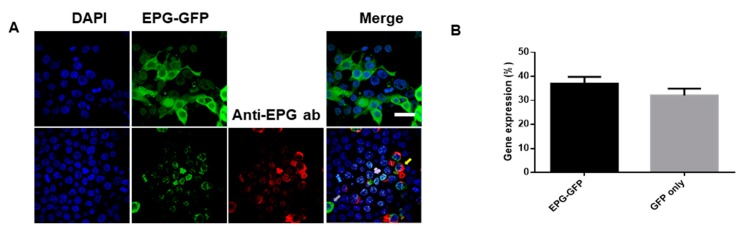
Fluorescence images of EPG expression in HEK293T cells. (**A**) EPG+GFP-transfected HEK293T cells (top images) and immunostaining of EPG+GFP expressed in HEK293T cells (bottom images). Primary antibodies: anti-EPG antibody from a rabbit, secondary antibodies: Alexa 595 conjugated anti-rabbit antibodies from a donkey. Yellow arrow = EPG+GFP with dye-conjugated anti-EPG antibody. Gray arrow = EPG+GFP. (Scale bar = 20 µm, DAPI, blue: 358⁄461 nm, GFP, green: 475/510 nm, Alexa 595, red: 540/595 nm). (**B**) Gene expression efficiency test (DNA; 2.5 ng/µL, Lipofectamine 2000:5 µL, n = 300).

The HEK293T cells were transfected with pcDNA3.1-EPG+GFP and fluorescent imaging was performed after maximal expression was observed. Figure 2A show that the expression of EPG was localized to the cell membrane. Immunofluorescence staining was performed using primary anti-EPG antibodies and a secondary Alexa-595 anti-rabbit antibodies. This confirmed that the EPG was fluorescently stained red and was expressed in the cell membrane [32] (Figure 2A). Figure 2B shows that approximately 40% of HEK293T cells express EPG.

### 3.3. Tagging EPG Expressing Cells with Magnetic Particles

**Figure 3:**
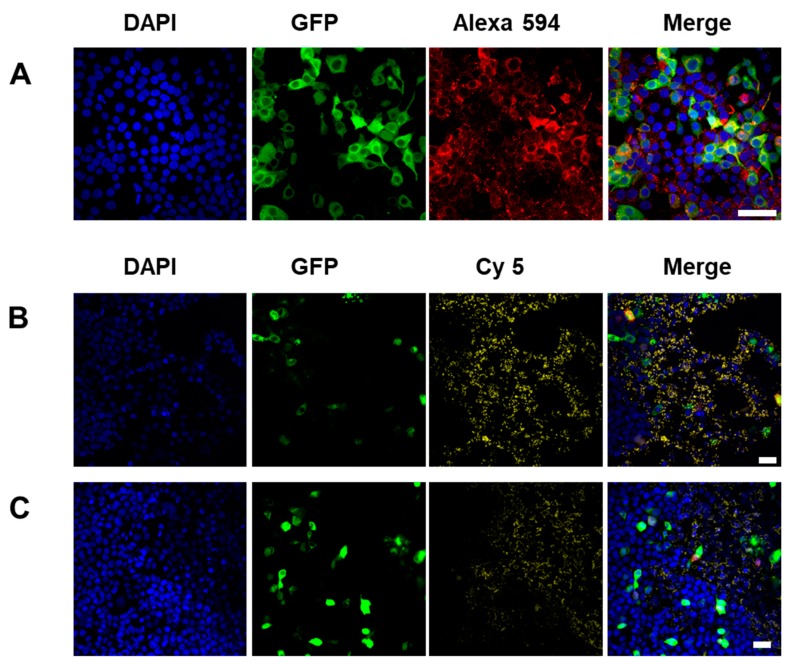
Images of HEK293T cells after the addition of magnetic particles. (**A**) Biotinylated HEK293T^EPG+GFP^ cells with Alexa 594-conjugated streptavidin (×40). (**B**) HEK293T^EPG+GFP^ cells with Cy5 conjugated ferromagnetic particles (×20). (**C**) HEK293T^EPG+GFP^ cells with Cy5 conjugated superparamagnetic nanoparticles (×20, Blue: DAPI, Green: EPG+GFP, Yellow: Cy5; scale bar = 20 μm).

In the previous study, we confirmed EPG activation by stimulation with either a 250 mT electromagnetic field (EMF) or a static magnet (25–50 mT) [32]. In this study we focused on fabricating an on/off switch that controls Ca^2+^ ion channels using MNPs and the intracellular EPG expression and Ca response using a minimal local magnetic field (0.2 mT). GFP expression which is independent of EPG, due to its dual transcription with IRES was localized to the cell cytoplasm (Figure 3A). It is important to note that the GFP is only indicative of the translation of the plasmid and not the localization of EPG. Here, biotin was introduced with Sulfo-NHS-biotin to the amine functional group present on the cell surface [35] and fluorescence imaging was performed by binding Alexa 594-streptavidin. Cell surface binding of streptavidin to biotion was confirmed (Figure 3A; in red). After Cy5-streptavidin-conjugated FMPs were added, the particles were uniformly distributed on the cell surface, as shown in Figure 3B. In addition, Cy5-streptavidin-conjugated SMNPs were similarly attached to the cells (Figure 3C).

### 3.4. Calcium Imaging

**Figure 4:**
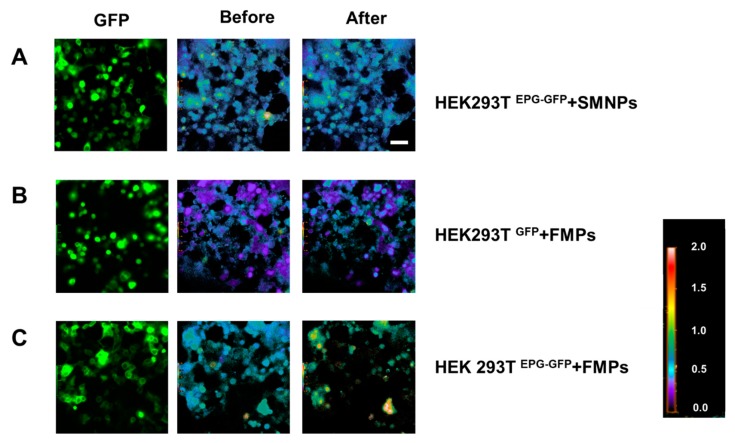
Calcium ion imaging following stimulation with different types of magnetic particles. Images of [Ca^2+^]_i_ in EPG+GFP-transfected HEK293T cells (the original GFP images: left column, 340/380 ratio pseudo-images before adding MPs: middle column, 340/380 nm ratio image after the addition of MPs: right column, 20×). (**A**) HEK293T^EPG+GFP^ with SMNPs. (**B**) HEK293T^GFP^ with Ferromagnetic particles (FMPs). (**C**) HEK293T^EPG+GFP^ with FMPs.

As shown in Figure 4, the signal intensity was increased when the FMPs were introduced to HEK293T^EPG+GFP^. However, when HEK293T^GFP^ was treated with the SMNPs, the signal intensity did not change. This result suggests that different types of magnetic particles can be integrated as on or off calcium ion switches. Fura-2-AM is a ratiometric fluorescent dye that binds to free intracellular calcium. Once inside the cell, esterase enzymes cleave the AM groups, leaving Fura-2-free acid trapped inside the cell where it binds to Ca^2+^ ions. It is excited at a ratio of 340/380 nm and emits fluorescence with a 510-nm peak [36,37]. Ca^2+^ fluorescent signal intensity before and after magnetic stimulation are shown for different conditions (Figure 4). When the SMNPs were added to the HEK293T^EPG+GFP^ cellsfluorescent intensity was not changed significantly (Figure 4A). When HEK293T^GFP^ were stimulated with the FMPs, a change in fluorescence due to the 340/380 nm ratio was not observed (Figure 4B). It implies that [Ca^2+^]_i_ was not changed with local magnetic stimulation due to the absence of the EPG in the cells. Microscopic images showed that the [Ca^2+^]_i_ of arbitrarily selected EPG+GFP positive cells significantly changed the 340/380 nm ratio due to FMP stimulation (Figure 4C; red circles). Although not all of the HEK293T^EPG+GFP^ cells responded to magnetic stimulation, the several cells showed that the concentration of the Ca^2+^ ion was changed by FMP stimulation. This result confirms that the switch (magnetic particles) works and the transfection and function of the transistor (EPG) are successful. 

### 3.5. FMPs Induce [Ca^2+^]_i_ Influx in Cells Expressing EPG

**Figure 5:**
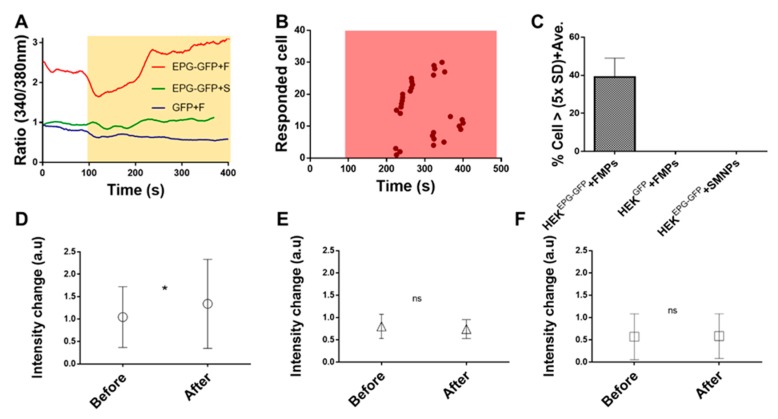
EPG-expressing HEK293T cell response to FMPs. (**A**) Representative [Ca^2+^]_i_ profiles depending on particle stimulation and gene expression (n = 1). Magnetic particles were added at 100 s, and Fura-2-AM dye was used to analyze [Ca^2+^]_i_ in EPG-positive cells. (**B**) The number of HEK cells responding to stimulation (n = 30, single cells were evaluated after the addition of magnetic particles). (**C**) A graph of EPG+GFP- and GFP-transfected cells that responded to FMPs and SMNPs. (**D**) Fluorescence intensity change of HEK293T^EPG+GFP^ cells by FMPs (Fura-2-AM-stained HEK293T^EPG+GFP^ cells). (**E**) HEK293T^GFP^ cells with FMPs. (**F**) HEK293T^EPG+GFP^ cells with SMNPs (n = 100). Non-significant values are shown as ns, while * describe the p value of <0.05.

In Figure 4A, the HEK293T^EPG+GFP^ + SMNPs showed a change in the 340/380 nm ratio of some cells. However, this was not significant. EPG-transfected cells activated an intracellular Ca ion increase that was significantl when assisted by selective magnetic particles. The HEK293T^EPG+GFP^ cells were treated with 10 µL of FMPs or SMNPs at 100 s and the unbound particles were removed by a silicon tubing system. When the FNPs were applied to the HEK293T^EPG+GFP^, an initial decrease in the fluorescence intensity was observed for 20 s after a interval of 100 s (Figure 5A). This time period is attributed to the absorption of light by the particles. A later increase in the fluorescence intensity (after 120 s) was due to the uptake of calcium into the cells. Unlike EPG-transduced neurons, response time is delayed in the HEK293T cells. This phenomenon should be studied further [32]. In Figure 5B, the reaction time of [Ca^2+^]_i_ was found to be between 120 s and 280 s following the addition of FMPs at 100 s. EPG-expressing cells were found to react within 180 s.

As a result, 40% ± 10% of the HEK293T^EPG+GFP^ cells with FMPs responded to magnetic stimulation (Figure 5C). In contrast, none of the HEK293T^EPG+GFP^ cells with SMNPs or the HEK293T^GFP^ cells with FMPs responded to magnetic stimulation (Figure 5C). These results confirm that intracellular Ca ion increased by a magnetic field more significantly in EPG-expressed cells when assisted by selective magnetic particles. For a more accurate statistical analysis, we compared the 340/380 nm ratio intensities of 100 single cells before and after treatment. Changes in [Ca^2+^]_i_ were only observed in the HEK293T^EPG+GFP^ cells (Figure 5D, * *p* < 0.05, Supplementary Video S1) stimulated with FMPs, but not in the HEK293T^EPG+GFP^ cells, stimulated with SMNPs or in the HEK293T^GFP^ cells stimulated with FMPs (Figure 5E,F, ns).

The magnetic particle cytotoxicity experiment confirmed that both SMNPs and FMPs were not cytotoxic at a concentration of 10 µL/mL that was used in the experiment (Supplementary Figure S2B).

The putative activation mechanism is represented in Figure 1. When a magnetic field is applied around the target cells, the magnetic particles promote the enhanced magnetic field around the target cells. Consequently, more EPGs are activated, leading to a local increase in the calcium concentration similar to the amplification of the calcium signal. However, further studies should carefully investigate whether the Ca^2+^ influx is from an external or internal release from the calcium reservoir.

## 4. Conclusions

Cells were stimulated by a small magnetic field of less than ~0.2mT and EPG stimulation was confirmed to modulate Ca^2+^ flux. The results of the present research confirm that the distance between the magnet and the cell is critical and further studies would verify the relationship. EPG-transfected cells activated an intracellular Ca^2+^ ion increase by a magnetic field more significantly when assisted by selective magnetic particles. It was confirmed by calcium imaging using FURA2AM dye. Here we report for the first time, the construction of a biological circuit using a combination of magnetic nanoparticles and a genetically encoded protein (EPG) stimulated by a magnetic field. Together, the EPG and the FMPs create a selective calcium-signal-stimulating system (similar to a transistor in an electronic circuit) that can potentially trigger calcium influx and thereby activate calcium-sensitive promoters such as c-fos, BDNF, or NFAT and consequently selectively express target proteins for use in various biomedical applications such as cardiac and/or neuroscience research. It is confirmed that the EPG is related to the control of Ca ion channels but the exact pathway is unknown. The proper design of gene sequence could be the key to responsiveness to EMP. Further studies regarding possible mechanisms should be performed.

## Supplementary Materials

The following are available online at https://www.mdpi.com/2218-273X/10/2/308/s1, Figure S1: Characterization of magnetic particles, Figure S2: Video of [Ca^2+^]i in EPG+GFP-transfected HEK 293T cells (x40, a heatmap is the same as in Figure 4, time scale: 1 s in video is 5 s), Video S1: [Ca^2+^]_i_ in cells.

## References

[1] Seo Y., Kim J.-E., Jeong Y., Lee K.H., Hwang J., Hong J., Park H., Choi J. (2016). Engineered nanoconstructs for the multiplexed and sensitive detection of high-risk pathogens. Nanoscale.

[2] Schultz B.R., Chamberlain J.S. (2008). Recombinant adeno-associated virus transduction and integration. Mol. Ther..

[3] Potenza L., Ubaldi L., De Sanctis R., De Bellis R., Cucchiarini L., Dachà M. (2004). Effects of a static magnetic field on cell growth and gene expression in Escherichia coli. Mutat. Res..

[4] Ventura C., Maioli M., Pintus G., Gottardi G., Bersani F. (2000). Elf-pulsed magnetic fields modulate opioid peptide gene expression in myocardial cells. Cardiovasc. Res..

[5] Hammond T., Lewis F., Goodwin T., Linnehan R., Wolf D., Hire K., Campbell W., Benes E., O’reilly K., Globus R. (1999). Gene expression in space. Nat. Med..

[6] Lewis M.L. (2002). The cytoskeleton, apoptosis, and gene expression in T lymphocytes and other mammalian cells exposed to altered gravity. Adv. Space Biol. Med..

[7] Oehler S., Eismann E.R., Krämer H., Müller-Hill B. (1990). The three operators of the lac operon cooperate in repression. EMBO J..

[8] Liu Q., Kasuga M., Sakuma Y., Abe H., Miura S., Yamaguchi-Shinozaki K., Shinozaki K. (1998). Two transcription factors, DREB1 and DREB2, with an EREBP/AP2 DNA binding domain separate two cellular signal transduction pathways in drought-and low-temperature-responsive gene expression, respectively, in Arabidopsis. Plant Cell.

[9] Ziyadeh F.N., Sharma K., Ericksen M., Wolf G. (1994). Stimulation of collagen gene expression and protein synthesis in murine mesangial cells by high glucose is mediated by autocrine activation of transforming growth factor-beta. J. Clin. Investig..

[10] Dalton T.P., Shertzer H.G., Puga A. (1999). Regulation of gene expression by reactive oxygen. Annu. Rev. Pharmacol. Toxicol..

[11] Takehara K., LeRoy E.C., Grotendorst G.R. (1987). TGF-β inhibition of endothelial cell proliferation: alteration of EGF binding and EGF-induced growth-regulatory (competence) gene expression. Cell.

[12] Ando H., Furuta T., Tsien R.Y., Okamoto H. (2001). Photo-mediated gene activation using caged RNA/DNA in zebrafish embryos. Nat. Genet..

[13] Schindler S.E., McCall J.G., Yan P., Hyrc K.L., Li M., Tucker C.L., Lee J.-M., Bruchas M.R., Diamond M.I. (2015). Photo-activatable Cre recombinase regulates gene expression in vivo. Sci. Rep..

[14] Gunaydin L.A., Yizhar O., Berndt A., Sohal V.S., Deisseroth K., Hegemann P. (2010). Ultrafast optogenetic control. Nat. Neurosci..

[15] Weber W., Lienhart C., Daoud-El Baba M., Grass R.N., Kohler T., Müller R., Stark W.J., Fussenegger M. (2009). Magnet-guided transduction of mammalian cells and mice using engineered magnetic lentiviral particles. J. Biotechnol..

[16] Miyakoshi J., Ohtsu S., Shibata T., Takebe H. (1996). Exposure to magnetic field (5 mT at 60 Hz) does not affect cell growth and c-myc gene expression. J. Radiat. Res..

[17] Akhavan O., Ghaderi E. (2013). Flash photo stimulation of human neural stem cells on graphene/TiO 2 heterojunction for differentiation into neurons. Nanoscale.

[18] Lavoie-Cardinal F., Salesse C., Bergeron É., Meunier M., De Koninck P. (2016). Gold nanoparticle-assisted all optical localized stimulation and monitoring of Ca 2+ signaling in neurons. Sci. Rep..

[19] Busskamp V., Picaud S., Sahel J.-A., Roska B. (2012). Optogenetic therapy for retinitis pigmentosa. Gene Ther..

[20] Meech R., Standen N. (1975). Potassium activation in Helix aspersa neurones under voltage clamp: a component mediated by calcium influx. J. Physiol..

[21] Barradas A.M., Fernandes H.A., Groen N., Chai Y.C., Schrooten J., van de Peppel J., van Leeuwen J.P., van Blitterswijk C.A., de Boer J. (2012). A calcium-induced signaling cascade leading to osteogenic differentiation of human bone marrow-derived mesenchymal stromal cells. Biomaterials.

[22] D’ascenzo M., Piacentini R., Casalbore P., Budoni M., Pallini R., Azzena G.B., Grassi C. (2006). Role of L-type Ca2+ channels in neural stem/progenitor cell differentiation. Eur. J. Neurosci..

[23] Itzhaki I., Rapoport S., Huber I., Mizrahi I., Zwi-Dantsis L., Arbel G., Schiller J., Gepstein L. (2011). Calcium handling in human induced pluripotent stem cell derived cardiomyocytes. PLoS One.

[24] Carbone E., Lux H. (1984). A low voltage-activated, fully inactivating Ca channel in vertebrate sensory neurones. Nature.

[25] Brewbaker J.L., Kwack B.H. (1963). The essential role of calcium ion in pollen germination and pollen tube growth. Am. J. Bot..

[26] Katz B., Miledi R. (1968). The role of calcium in neuromuscular facilitation. J. Physiol..

[27] McCormack J.G., Halestrap A.P., Denton R.M. (1990). Role of calcium ions in regulation of mammalian intramitochondrial metabolism. Physiol. Rev..

[28] Farber J.L. (1981). The role of calcium in cell death. Life Sci..

[29] Striggow F., Ehrlich B.E. (1996). Ligand-gated calcium channels inside and out. Curr. Opin. Cell Biol..

[30] Burnashev N. (1998). Calcium permeability of ligand-gated channels. Cell Calcium.

[31] Oertner T.G., Single S., Borst A. (1999). Separation of voltage-and ligand-gated calcium influx in locust neurons by optical imaging. Neurosci. Lett..

[32] Krishnan V., Park S.A., Shin S.S., Alon L., Tressler C.M., Stokes W., Banerjee J., Sorrell M.E., Tian Y., Fridman G.Y. (2018). Wireless control of cellular function by activation of a novel protein responsive to electromagnetic fields. Sci. Rep..

[33] Wohlfarth E.P. (1958). Relations between different modes of acquisition of the remanent magnetization of ferromagnetic particles. J. Appl. Phys..

[34] Mørup S., Tronc E. (1994). Superparamagnetic relaxation of weakly interacting particles. Phys. Rev. Lett..

[35] Hwang J., Seo Y., Jo Y., Son J., Choi J. (2016). Aptamer-conjugated live human immune cell based biosensors for the accurate detection of C-reactive protein. Sci. Rep..

[36] Roe M., Lemasters J., Herman B. (1990). Assessment of Fura-2 for measurements of cytosolic free calcium. Cell Calcium.

[37] Karaki H., Ozaki H., Hori M., Mitsui-Saito M., Amano K.-I., Harada K.-I., Miyamoto S., Nakazawa H., Won K.-J., Sato K. (1997). Calcium movements, distribution, and functions in smooth muscle. Pharmacol. Rev..

